# Supplementary material for: Trapping and detecting nanoplastics by MXene-derived oxide microrobots
Source: Nat Commun. 2022 Jun 22;13:3573. doi: 10.1038/s41467-022-31161-2 (PMC9218121; doi:10.1038/s41467-022-31161-2)
Supplement: Supplementary file 1 — Supplementary Information [file 41467_2022_31161_MOESM1_ESM.pdf]

Supplementary Information

Trapping and detecting nanoplastics by MXene-derived oxide microrobots

Mario Urso et. al.

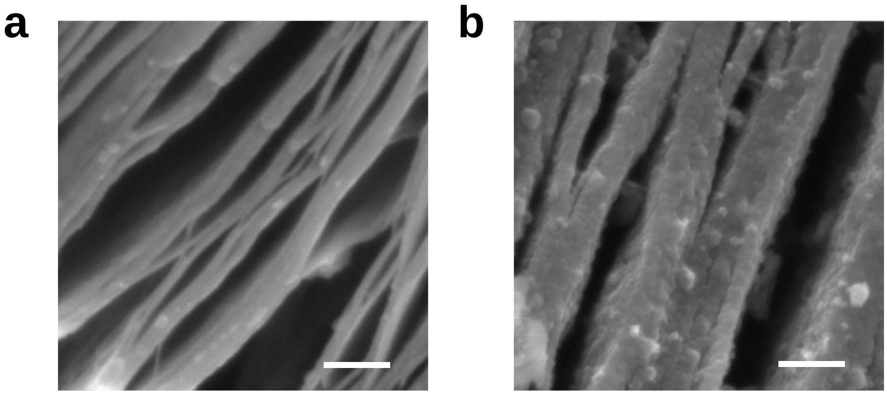

Supplementary Figure 1. High-resolution SEM images. **a** $Ti_3C_2T_x$ MXene microparticle and **b** MXene-derived γ-Fe_2O_3/Pt/TiO_2 microrobot (thermal annealing condition: 0 min at 550°C). Scale bars are 200 nm.

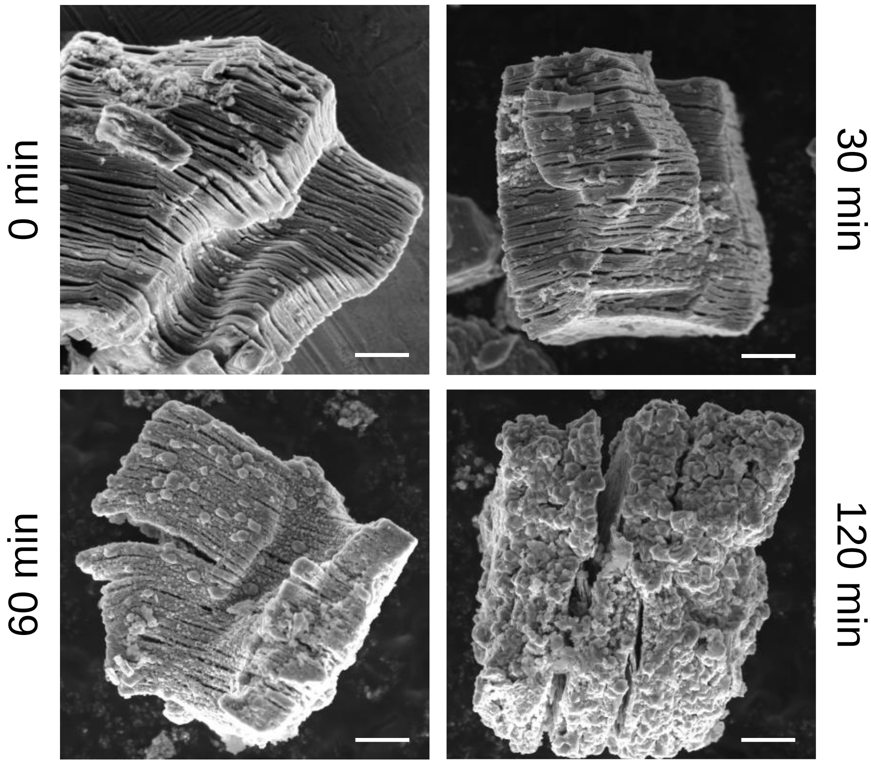

Supplementary Figure 2. SEM images of MXene-derived TiO_2 microparticles obtained by thermal annealing in air at 550°C for 0, 30, 60, and 120 min. Scale bars are 2 μm.

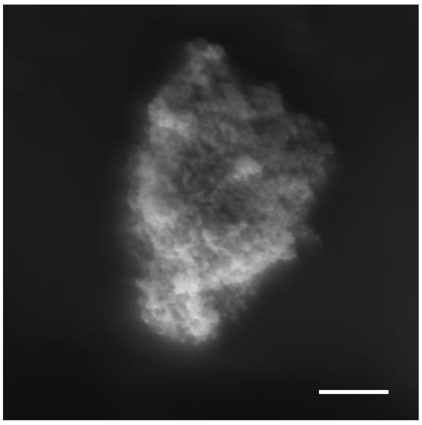

Supplementary Figure 3. SEM image of a cluster of γ-Fe_2O_3 nanoparticles. The scale bar is 500 nm.

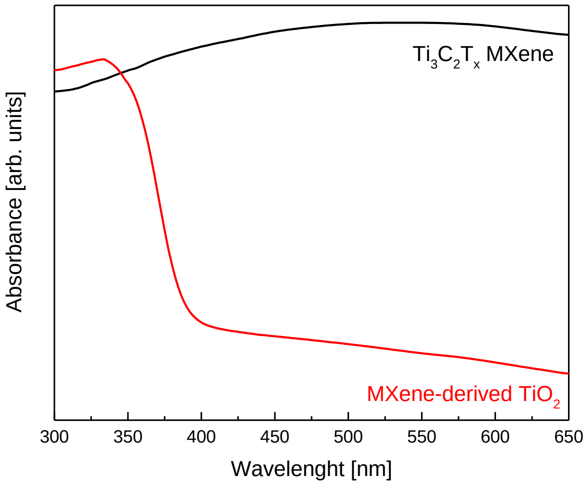

Supplementary Figure 4. Light absorption spectra of $Ti_3C_2T_x$ MXene and MXene-derived TiO_2 microparticles (thermal annealing condition: 0 min at 550°C).

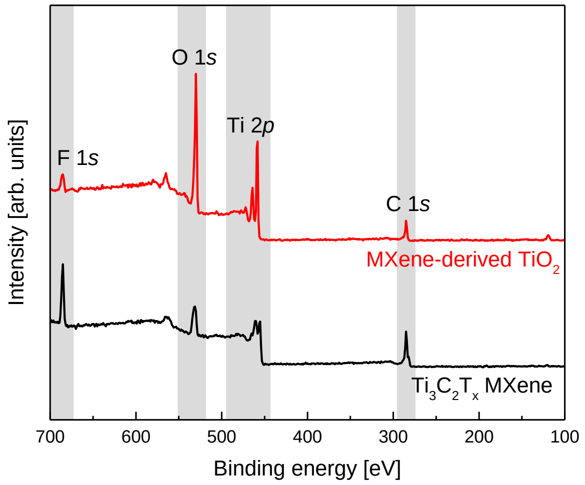

Supplementary Figure 5. XPS survey spectra of $Ti_3C_2T_x$ MXene and MXene-derived TiO_2 microparticles (thermal annealing condition: 0 min at 550°C).

Supplementary Table 1. XPS peak fitting results for $Ti_3C_2T_x$ MXene microparticles, MXene-derived TiO_2 microparticles, and MXene-derived γ-Fe_2O_3/Pt/TiO_2 microrobots (thermal annealing condition: 0 min at 550°C).

Sample	Region	Binding energy [eV]	Assigned to	Ref.
$Ti_3C_2T_x$ MXene	Ti 2$p_{3/2}$ (Ti 2$p_{1/2}$)	454.9 (460.5)	Ti–C (MXene)	1
		455.8 (461.3)	Ti^{2+}	
		456.9 (462.5)	Ti^{3+}	
		458.9 (464.6)	TiO_2	
		459.4 (465.4)	$TiO_{2-x}F_x$	
	C 1*s*	281.7	C–Ti–T_x	1
		284.8	C–C	
		286.3	CH_x/C–O	
		288.9	O–C=O	2
	O 1*s*	529.9	TiO_2	1
		531.2	C-Ti-O_x	
		532.0	C-Ti-$(OH)_x$	
		533.8	H_2O_{ads}	
MXene-derived TiO_2	Ti 2*p*	458.6	Ti 2$p_{3/2}$	1
		464.3	Ti 2$p_{1/2}$	
	C 1*s*	285.0	C–C	1
		286.5	C–O–C	2
		289.0	O–C=O	
	O 1*s*	529.8	TiO_2	1
		531.4	C–Ti–O_x	
MXene-derived γ-Fe_2O_3/Pt/TiO_2 microrobots	Pt 4*f*	71.0	Pt^0 4$f_{7/2}$	3
		74.3	Pt^0 4$f_{5/2}$	
	Fe 2$p_{3/2}$ (Fe 2$p_{1/2}$)	710.8 (723.9)	Peak 1	4
		712.9 (726.0)	Peak 2	
		719.4 (732.5)	Satellite	

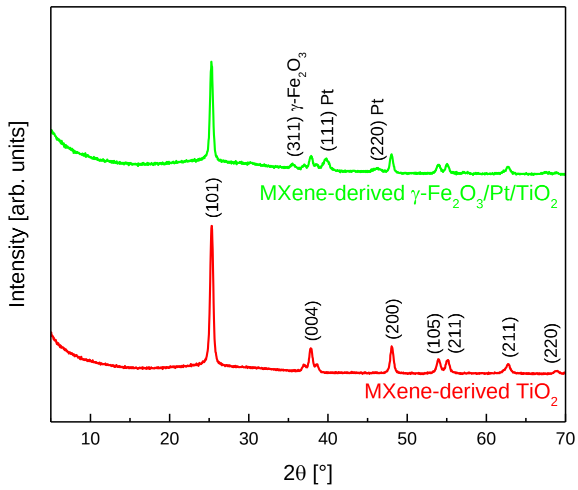

Supplementary Figure 6. XRD patterns of the MXene-derived TiO_2 microparticles and MXene-derived γ-Fe_2O_3/Pt/TiO_2 microrobots (thermal annealing condition: 0 min at 550°C).

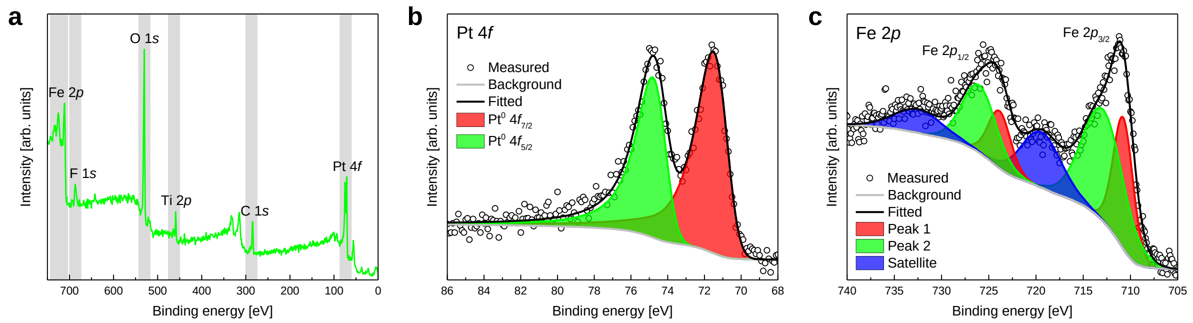

Supplementary Figure 7. XPS analysis of the MXene-derived γ-Fe_2O_3/Pt/TiO_2 microrobots (thermal annealing condition: 0 min at 550°C). **a** XPS survey spectrum. **b** Pt 4*f* and **c** Fe 2*p* high-resolution XPS spectra.

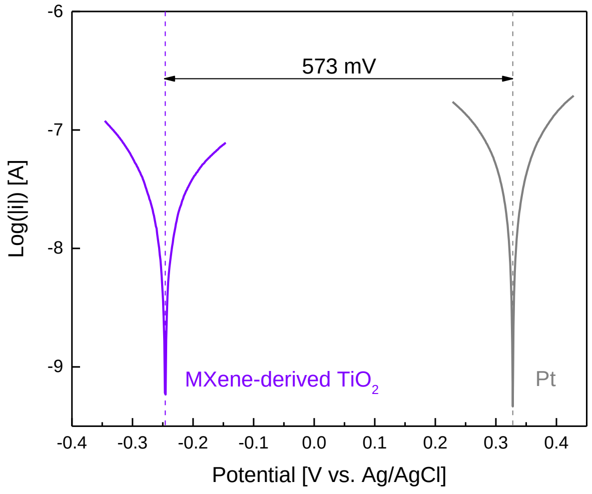

Supplementary Figure 8. Tafel plots of MXene-derived TiO_2 and Pt electrodes under UV-light irradiation in ultra-pure water.

Supplementary Discussion 1

To allow a straightforward comparison with nanoplastics' concentration values measured through nanoparticle tracking analysis (NTA), the nanoplastics' concentration (nanoplastics ml^{-1}) in the commercial suspension before any dilution was calculated as follows. According to the provider, the suspension consists of carboxylated polystyrene nanoparticles in water with a concentration (c) of 10 mg ml^{-1}. The nanoparticles have an average diameter of 50 nm, which was also confirmed by NTA. The volume (V) of a spherical particle with radius r is $V = \frac{4}{3}\pi r^3$. Therefore, a volume of 65.4×10^{-18} cm^3 was calculated for the single nanoplastic. Then, given a polystyrene density (ρ) of 1.05 g cm^{-3}, the mass (m) of the single nanoplastic was calculated to be 68.7×10^{-18} g through the relation $m = \rho V$. Finally, the nanoplastics' concentration was obtained from the ratio c/m, finding an approximate value of 1.5×10^{14} nanoplastics ml^{-1}.

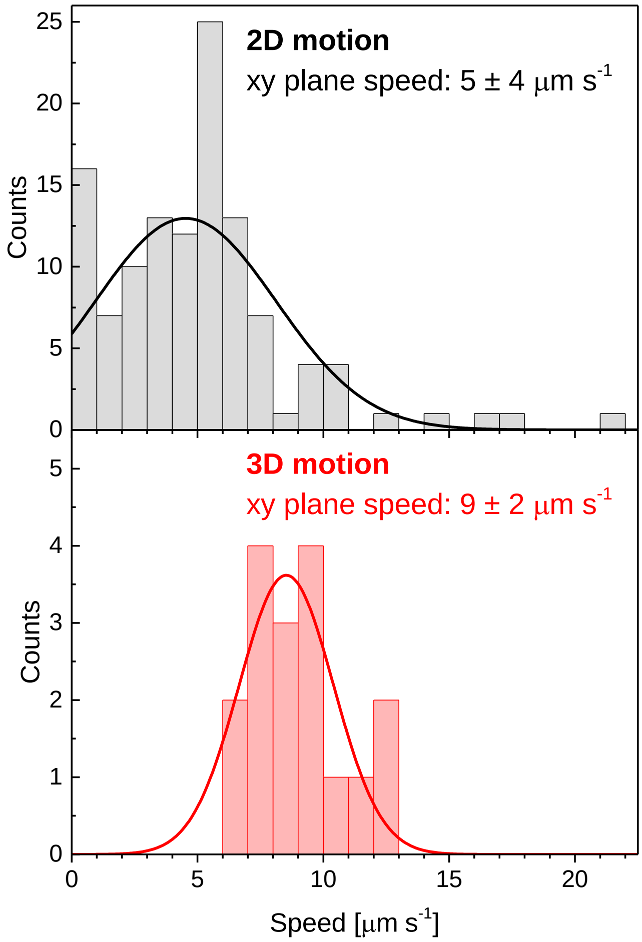

Supplementary Figure 9. Speed distributions of the MXene-derived γ-Fe_2O_3/Pt/TiO_2 microrobots (thermal annealing condition: 0 min at 550°C) showing 2D and 3D motion in fuel-free water at pH 3 under UV-light irradiation.

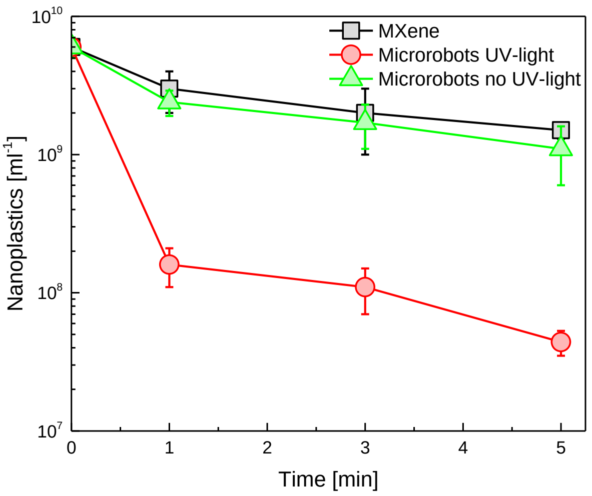

Supplementary Figure 10. Nanoplastics' concentration in the treated suspensions as a function of the treatment's duration (0, 1, 3, and 5 min) using γ-$Fe_2O_3/Ti_3C_2T_x$ MXene microparticles under UV-light irradiation, and MXene-derived γ-Fe_2O_3/Pt/TiO_2 microrobots (thermal annealing condition: 0 min at 550°C) with and without UV-light irradiation (other capture experiments conditions: 0.75 mg ml^{-1} samples, 6x10^9 nanoplastics ml^{-1}, water at pH 3, samples' collection using a neodymium magnet). Error bars represent the standard deviation, n = 3 independent replicates.

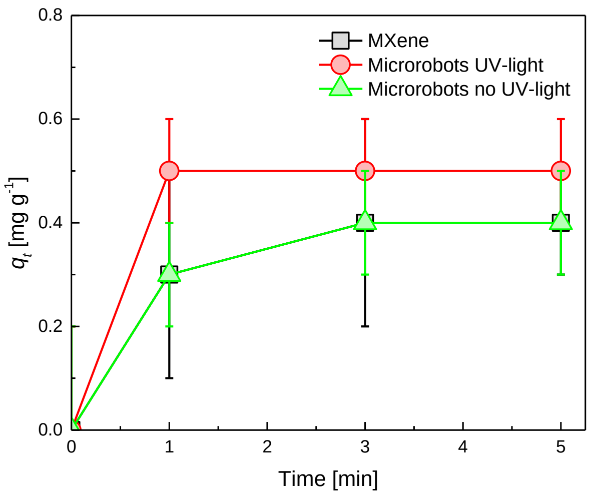

Supplementary Figure 11. Removal capacity q_t, calculated through Equation (1) in the manuscript, as a function of the treatment's duration (0, 1, 3, and 5 min) using γ-$Fe_2O_3/Ti_3C_2T_x$ MXene microparticles under UV-light irradiation, MXene-derived γ-Fe_2O_3/Pt/TiO_2 microrobots (thermal annealing condition: 0 min at 550°C) with and without UV-light irradiation (other capture experiments conditions: 0.75 mg ml^{-1} samples, 6x10^9 nanoplastics ml^{-1}, water at pH 3, samples' collection using a neodymium magnet). Error bars represent the standard deviation, n = 3 independent replicates.

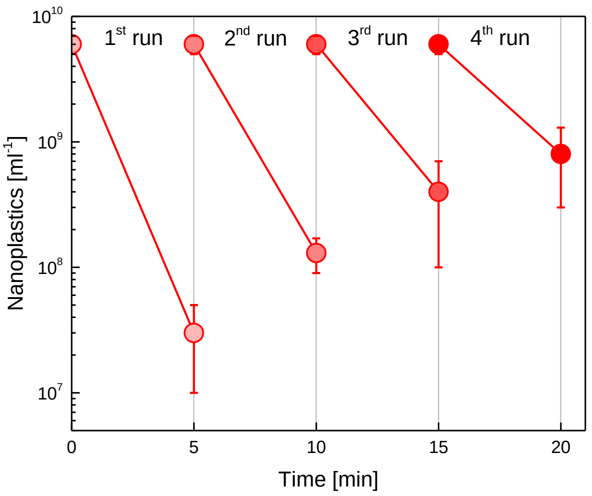

Supplementary Figure 12. Reusability test using the MXene-derived γ-Fe_2O_3/Pt/TiO_2 microrobots (thermal annealing condition: 0 min at 550°C). Capture experiments conditions: 0.75 mg ml^{-1} microrobots, 6x10^9 nanoplastics ml^{-1}, water at pH 3, UV-light irradiation for 5 min, microrobots collection using a neodymium magnet. After each run, the captured nanoplastics were released under vigorous agitation in water at pH 11 for 10 min. Error bars represent the standard deviation, n = 3 independent replicates.

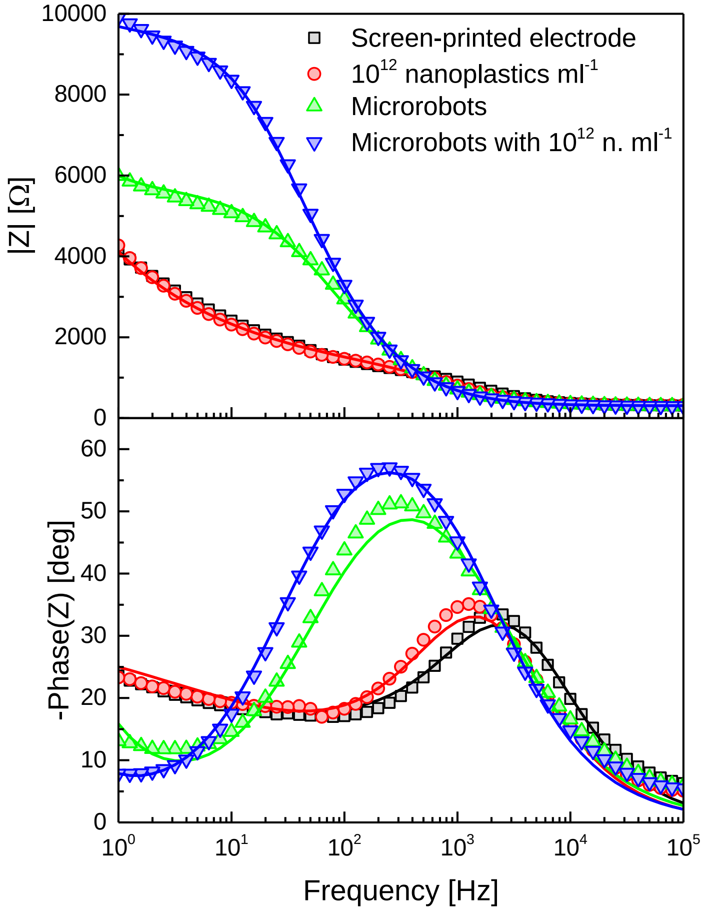

Supplementary Figure 13. Bode plots for nanoplastics' detection by electrochemical impedance spectroscopy (EIS) technique after the preconcentration with the MXene-derived γ-Fe_2O_3/Pt/TiO_2 microrobots. The plots show the impedance modulus (|Z|) and phase (-Phase(Z)) as a function of the frequency for a bare screen-printed electrode (SPE), an SPE exposed to ~10^{12} nanoplastics ml^{-1} suspension, an SPE after MXene-derived γ-Fe_2O_3/Pt/TiO_2 microrobots' (thermal annealing condition: 0 min at 550°C) loading, and an SPE after loading microrobots with captured nanoplastics (capture experiment conditions: 0.75 mg ml^{-1} microrobots, ~10^{12} nanoplastics ml^{-1}, water at pH 3, UV-light irradiation for 1 min, microrobots collection using a neodymium magnet). The EIS measurements were performed in a 10 mM $Fe(CN)_6^{4-/3-}$ aqueous solution at 0 V vs. ref. with a 10 mV superimposed sinusoidal root-mean-square voltage in the frequency range $10^5 \div 10^0$ Hz. The lines represent fits to the data based on the equivalent circuit model in Figure 7(b) in the manuscript.

Supplementary Table 2. EIS fitting results based on the equivalent circuit model reported in Figure 7(b).

Sample	R_s [Ω]	R_{ct} [Ω]	Q_1 [μMho s^n]	n_1	Q_2 [μMho s^n]	n_2
Screen-printed electrode	305 ± 4	605 ± 77	0.27 ± 0.07	0.92 ± 0.03	165 ± 4	0.33 ± 0.01
10^{12} nanoplastics ml^{-1}	321 ± 4	813 ± 71	0.45 ± 0.09	0.91 ± 0.02	162 ± 5	0.37 ± 0.02
Microrobots	295 ± 4	5459 ± 130	1.7 ± 0.1	0.792 ± 0.009	120 ± 30	0.9 ± 0.1
Microrobots with 10^6 nanoplastics ml^{-1}	292 ± 2	5242 ± 205	0.98 ± 0.05	0.827 ± 0.005	173 ± 8	0.38 ± 0.04
Microrobots with 10^{12} nanoplastics ml^{-1}	203 ± 3	9480 ± 176	1.33 ± 0.06	0.829 ± 0.006	200 ± 100	0.9 ± 0.2
Microrobots with 10^{14} nanoplastics ml^{-1}	479 ± 3	18232 ± 547	5.7 ± 0.2	0.752 ± 0.004	80 ± 50	1.1 ± 0.3

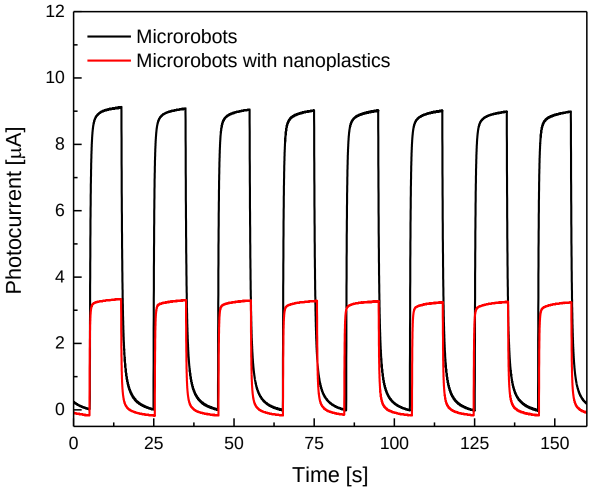

Supplementary Figure 14. Transient photocurrent response of MXene-derived γ-Fe_2O_3/Pt/TiO_2 microrobots (thermal annealing condition: 0 min at 550°C) before and after capturing nanoplastics (capture experiment conditions: 0.75 mg ml^{-1} microrobots, ~10^{12} nanoplastics ml^{-1}, water at pH 3, UV-light irradiation for 5 min, microrobots collection using a neodymium magnet). The photocurrent was measured at 0 V vs. OCP by turning on and off the UV-light irradiation at time intervals of 10 s.

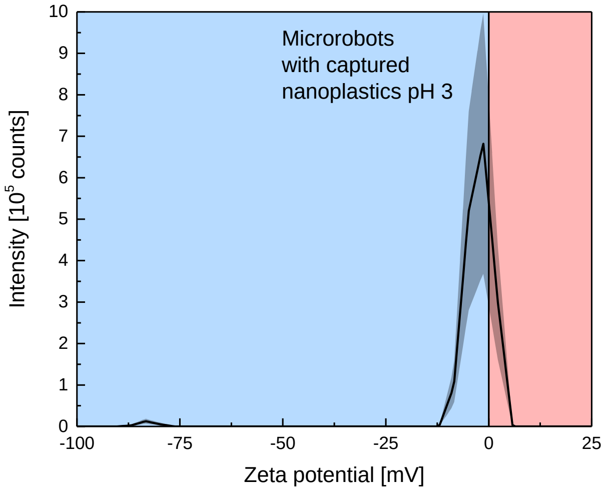

Supplementary Figure 15. Zeta potential of the MXene-derived γ-Fe_2O_3/Pt/TiO_2 microrobots (thermal annealing condition: 0 min at 550°C) after nanoplastics' capture in water at pH 3 (capture experiment conditions: 0.75 mg ml^{-1} microrobots, ~10^{12} nanoplastics ml^{-1}, water at pH 3, UV-light irradiation for 1 min, microrobots collection using a neodymium magnet). The shaded region replaces the discrete error bars calculated as the standard error for n = 3 independent replicates.

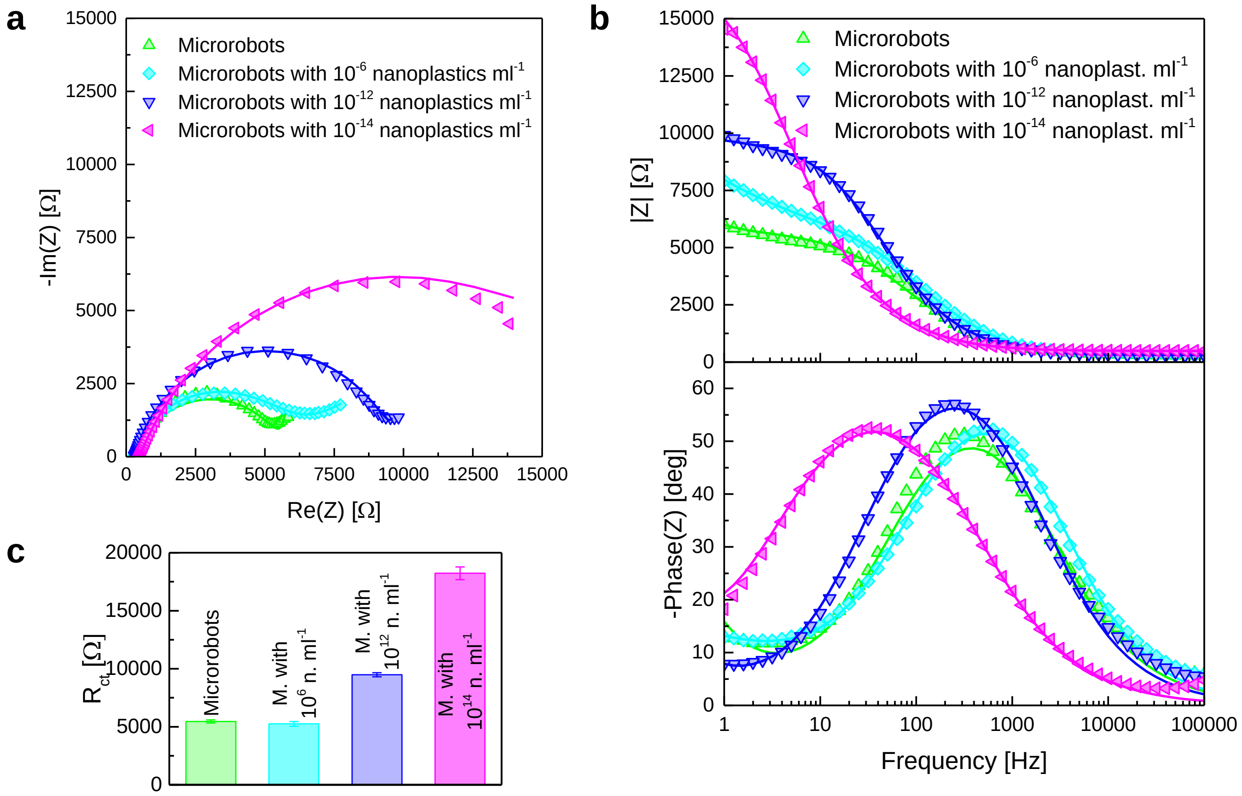

Supplementary Figure 16. Detection of nanoplastics at different concentrations by electrochemical impedance spectroscopy (EIS) technique after the preconcentration with the MXene-derived γ-Fe_2O_3/Pt/TiO_2 microrobots. **a** Nyquist plots showing the impedance real (Re(Z)) and imaginary (-Im(Z)) parts as a function of the frequency and **b** Bode plots showing the impedance modulus (|Z|) and phase (-Phase(Z)) as a function of the frequency for a screen-printed electrode (SPE) after MXene-derived γ-Fe_2O_3/Pt/TiO_2 microrobots' (thermal annealing condition: 0 min at 550°C) loading, and SPEs after loading microrobots with captured nanoplastics at different concentrations (capture experiment conditions: 0.75 mg ml^{-1} microrobots, ~10^6, 10^{12}, and 10^{14} nanoplastics ml^{-1}, water at pH 3, UV-light irradiation for 1 min, microrobots collection using a neodymium magnet). The EIS measurements were performed in a 10 mM $Fe(CN)_6^{4-/3-}$ aqueous solution at 0 V vs. ref. with a 10 mV superimposed sinusoidal root-mean-square voltage in the frequency range $10^5 \div 10^0$ Hz. The lines represent fits to the data based on the equivalent circuit model reported in Figure 7(b) in the manuscript. **c** R_{ct} values obtained from the Nyquist plots fit. Error bars represent the fitting error.

Supplementary References

1. Halim, J. *et al.* X-ray photoelectron spectroscopy of select multi-layered transition metal carbides (MXenes). *Appl. Surf. Sci.* **362**, 406–417 (2016).

2. Huang, Y. L. *et al.* Effect of extended polymer chains on properties of transparent graphene nanosheets conductive film. *J. Mater. Chem.* **21**, 18236–18241 (2011).

3. Bera, P. *et al.* Ionic dispersion of Pt over CeO_2 by the combustion method: Structural investigation by XRD, TEM, XPS, and EXAFS. *Chem. Mater.* **15**, 2049–2060 (2003).

4. Biesinger, M. C. *et al.* Resolving surface chemical states in XPS analysis of first row transition metals, oxides and hydroxides: Cr, Mn, Fe, Co and Ni. *Appl. Surf. Sci.* **257**, 2717–2730 (2011).
