# Supplementary material for: Trapping and detecting nanoplastics by MXene-derived oxide microrobots
Source: Nat Commun. 2022 Jun 22;13:3573. doi: 10.1038/s41467-022-31161-2 (PMC9218121; doi:10.1038/s41467-022-31161-2)
Supplement: Supplementary file 3 — Description of Additional Supplementary Files [file 41467_2022_31161_MOESM3_ESM.docx]

**Description of additional supplementary files**

**Supplementary Movie 1.** Magnetic collection of the MXene-derived γ-Fe_2_O_3_/Pt/TiO_2_ microrobots in water using a neodymium magnet.

**Supplementary Movie 2.** 2D motion of the MXene-derived γ-Fe_2_O_3_/Pt/TiO_2_ microrobots in fuel-free water under UV-light irradiation.

**Supplementary Movie 3.** 3D motion of the MXene-derived γ-Fe_2_O_3_/Pt/TiO_2_ microrobots in fuel-free water under UV-light irradiation.

**Supplementary Movie 4.** Self-orientation of an MXene-derived γ-Fe_2_O_3_/Pt/TiO_2_ microrobot showing 3D motion in fuel-free water upon switching on the UV-light irradiation.

**Supplementary Movie 5.** Video clips of nanoplastics’ suspensions after serial dilution (dilution factors: 5x10^4^, 1x10^5^, 5x10^5^, 1x10^6^, 5x10^6^, 1x10^7^) for nanoparticle tracking analysis (NTA).

**Supplementary Movie 6.** Video clips of nanoplastics’ suspensions after the treatment with γ-Fe_2_O_3_/Ti_3_C_2_T_x_ Mxene microparticles (“MXene”) and MXene-derived γ-Fe_2_O_3_/Pt/TiO_2_ microrobots (“Microrobots”) for nanoparticle tracking analysis (NTA). Capture experiments conditions: 0.75 mg ml^-1^ sample, 6x10^9^ nanoplastics ml^-1^, water at pH 3, samples’ collection using a neodymium magnet.
